# Multistability of bursting rhythms in a half-center oscillator and the protective effects of synaptic inhibition

**DOI:** 10.3389/fncel.2024.1395026

**Published:** 2024-09-17

**Authors:** Parker J. Ellingson, Yousif O. Shams, Jessica R. Parker, Ronald L. Calabrese, Gennady S. Cymbalyuk

**Affiliations:** ^1^Neuroscience Institute, Georgia State University, Atlanta, GA, United States; ^2^Department of Biology, Emory University, Atlanta, GA, United States

**Keywords:** bistability, seizure-like activity, plateau, neuromodulation, Na+/K+ pump current

## Abstract

For animals to meet environmental challenges, the activity patterns of specialized oscillatory neural circuits, central pattern generators (CPGs), controlling rhythmic movements like breathing and locomotion, are adjusted by neuromodulation. As a representative example, the leech heartbeat is controlled by a CPG driven by two pairs of mutually inhibitory interneurons, heart interneuron (HN) half-center oscillators (HCO). Experiments and modeling indicate that neuromodulation of HCO navigates this CPG between dysfunctional regimes by employing a co-regulating inverted relation; reducing Na^+^/K^+^ pump current and increasing hyperpolarization-activated (h-) current. Simply reducing pump activity or increasing h-current leads to either seizure-like bursting or an asymmetric bursting dysfunctional regime, respectively. Here, we demonstrate through modeling that, alongside this coregulation path, a new bursting regime emerges. Both regimes fulfill the criteria for functional bursting activity. Although the cycle periods and burst durations of these patterns are roughly the same, the new one exhibits an intra-burst spike frequency that is twice as high as the other. This finding suggests that neuromodulation could introduce additional functional regimes with higher spike frequency, and thus more effective synaptic transmission to motor neurons. We found that this new regime co-exists with the original bursting. The HCO can be switched between them by a short pulse of excitatory or inhibitory conductance. In this domain of coexisting functional patterns, an isolated cell model exhibits only one regime, a severely dysfunctional plateau-containing, seizure-like activity. This aligns with widely reported notion that deficiency of inhibition can cause seizures and other dysfunctional neural activities. We show that along the coregulation path of neuromodulation, the high excitability of the single HNs induced by myomodulin is harnessed by mutually inhibitory synaptic interactions of the HCO into the functional bursting pattern.

## 1 Introduction

Rhythmic circuits, called central pattern generators (CPGs), that control oscillatory motor behaviors like walking, swimming, and breathing, demonstrate a remarkable ability to produce patterns that are robust and, at the same time, can flexibly adjust to changes of the environment and behavioral goals (Marder and Calabrese, 1996; Calabrese, 1998; Doi and Ramirez, 2008; Marder, 2012; Alonso and Marder, 2020; Stadele and Stein, 2022). The neuromodulation making these adjustments does so by producing changes in membrane and synaptic currents often with seemingly opposing effects in a complex non-intuitive manner (Brezina et al., 2003a,b; Brezina et al., 2005; Harris-Warrick and Johnson, 2010; Ellingson et al., 2021). How the bursting neurons of these rhythmic circuits, which inherently display a wide range of biophysical parameters of ionic currents (Goldman et al., 2001; Prinz et al., 2003, 2004; Ellingson et al., 2021) respond to neuromodulation and deliver functional activity patterns with a wide range of the cycle period and spike frequency is not well understood.

The complexity of this imposition of neuromodulation on an inherently variable background, stems from its non-linear dynamical nature. Neurons and neuronal networks can exhibit different activity regimes, e.g., silence, spiking, or bursting, and their co-existence (Guttman et al., 1980; Hounsgaard et al., 1984; Hounsgaard and Kiehn, 1989; Lechner et al., 1996; Perrier and Hounsgaard, 2000; Williams et al., 2002; Loewenstein et al., 2005; Paydarfar et al., 2006; Newman and Butera, 2010; Forrest et al., 2012) only one of which may be functionally appropriate in a given context. Transitions between these regimes set limits to the working range of possible control by neuromodulators. Moreover, neuromodulation can be associated with multistability of activity regime. Multistability refers to the co-existence of more than one regimes in the phase space of the neuronal system. Neurons and their circuits can show a switch between regimes in response to short transient signals or transition between patterns in a state-dependent fashion, demonstrating hysteresis, which is the hallmark of multistability (Canavier et al., 1994; Lechner et al., 1996; Butera, 1998; Nadim et al., 2008; Newman and Butera, 2010; Malashchenko et al., 2011a,b; Marin et al., 2013; Parker et al., 2018, 2021). As a product of neuromodulation, multistability can be functional and desirable or dysfunctional and dangerous. Multistable neurons may play a role as toggle switches and memory units in information processing and working memory (Turrigiano et al., 1996; Fall et al., 2005; Loewenstein et al., 2005; Nadim et al., 2008; Forrest et al., 2012) and could be useful elements of multifunctional central pattern generators (Parker et al., 2018, 2021), but multistability can also be a pathological feature. For example, normal regimes can coexist with seizure regimes (Hahn and Durand, 2001; Frohlich and Bazhenov, 2006; Ziburkus et al., 2006; Cressman et al., 2009; Ullah et al., 2009).

In our previous work, we reported on how myomodulin in a half-center oscillator (HCO) of the leech heartbeat central pattern generator comodulates the h-current and the Na^+^/K^+^ pump current, coordinately increasing the former and decreasing the latter, to control the period of activity in a wide range while avoiding dysfunctional regimes (Ellingson et al., 2021). As the most dangerous dysfunctional regime, we considered the plateau-containing regimes observed at the low values of the maximal pump activity. Similar regimes have been described in leech neurons; widespread seizure-like plateau oscillations are induced by substituting external Ca^2+^ with blocking ions and these plateaus are terminated by pump activity (Angstadt and Friesen, 1991; Opdyke and Calabrese, 1994). The reduction of Na^+^/K^+^ pump activity has been also implicated in the transition to seizure-like plateau-containing activity in principal neurons of mouse hippocampal slices (Krishnan et al., 2015). During seizures the Na^+^/K^+^ pump appears to play a crucial role in termination and in postictal depression (Frohlich and Bazhenov, 2006, Ziburkus et al., 2006, Cressman et al., 2009; Ullah et al., 2009, Krishnan et al., 2015).

We hypothesized that given the prevalence of plateaus at low values of maximal pump current, *I_PumpMax_*, in our leech heart interneuron (HN) HCO model, the half-center structure may protect the system from succumbing to underlying pathological single cell dynamics. We compare maps of activity regimes of HCO model with a single HN model. Recently, we discovered that a single HN with augmented dynamics by upregulating persistent sodium and pump currents with dynamic clamp can produce a new type of bursting with high spike frequency and high amplitude of the underlying voltage envelope (Erazo-Toscano et al., 2023). Reviewing the waveforms of the HCO bursting activity along the myomodulin coregulation path (Ellingson et al., 2021), we noticed a change in the waveform and spike frequency suggesting emergence of a new bursting regime. Here, we present the discovery of a unique, functional multistable domain in an HCO model under modulated conditions and investigate the parameter space of modulation in a single cell HN model. We found that, in HCO, a high-spike-frequency high-voltage-amplitude bursting coexists with a low-spike-frequency low-voltage-amplitude bursting. The cycle period of these two regimes was the same. We found that the multistability of the two regimes appears in the HCO model and is not found in the decoupled HN model. We compare contributions of specific currents to generation of the two bursting rhythms. We describe several regimes and demonstrate how the HCO structure prevents the breakdown of functional activity under modulated conditions.

## 2 Materials and methods

### 2.1 Model and parameter sweeps

We have incorporated the Na^+^/K^+^ pump dynamics into the canonical model of the leech heart interneuron (Kueh et al., 2016; Ellingson et al., 2021; Toscano et al., 2021; Erazo-Toscano et al., 2023). The model consists of either a single neuron (single HN model) or two reciprocally inhibitory identical neurons (HCO model). It replicates the electrical activity of the HNs of the leech heartbeat CPG under a variety of experimental conditions (Opdyke and Calabrese, 1994; Olsen and Calabrese, 1996; Cymbalyuk and Calabrese, 2000; Cymbalyuk et al., 2002; Sorensen et al., 2004; Olypher et al., 2006; Tobin and Calabrese, 2006; Weaver et al., 2010; Kueh et al., 2016; Ellingson et al., 2021; Toscano et al., 2021). The individual HN was modeled as a single isopotential compartment with Hodgkin-Huxley type intrinsic membrane currents. It has 8 voltage-gated currents: fast Na^+^ current, I_NaF_; persistent Na^+^ current, I_P_; fast and slow low-threshold Ca^2+^ currents, I_CaF_ and I_CaS_; h-current, I_h_; delayed rectifier-like K^+^ current, I_K1_; a persistent K^+^ current, I_K2_; and a fast-transient K^+^ current, I_KA_. The HCO model includes inhibitory spike-mediated synaptic current. The HCO model is utilized as presented in Ellingson et al. (2021), while the single HN model uses the same set of equations without synapses. Parameter sweeps for the single cell model are conducted in the same manner as described in Ellingson et al. (2021).

### 2.2 Modeling the Na^+^/K^+^ pump

The activity of the pump depends on the extracellular K^+^ concentration ( ${\left[K\right]}_{o}$ ) and the intracellular Na^+^ concentration ( ${\left[Na\right]}_{i}$ ). We assume that the external and internal K^+^, and external Na^+^ concentrations do not change significantly during normal operation of heart interneurons and that the pump rate is not voltage dependent. We model the pump current following Angstadt and Friesen (1991) and Kueh et al. (2016).

$I_{\mathrm{pump}}=\frac{I_{\mathrm{PumpMax}}}{1+\exp\left(\frac{{\left[Na\right]}_{ih}-{\left[Na\right]}_{i}}{{\left[Na\right]}_{\mathrm{is}}}\right)}$ , where $I_{\mathrm{PumpMax}}$ is the maximal pump current, ${\left[Na\right]}_{ih}$ is the internal concentration of Na^+^ at which the steady state rate of the pump is one half of the maximal value; ${\left[Na\right]}_{\mathrm{is}}$ determines the responsivity of the pump to changes of ${\left[Na\right]}_{i}$ : the smaller the value of ${\left[Na\right]}_{\mathrm{is}}$ is, the steeper is the pump steady state activation curve and the more responsive is the pump current.

The two sodium currents (I_NaF_ and I_P_), I_h_, I_leak_ and the pump current determine ${\left[Na\right]}_{i}$ . I_h_ is a mixed cation current carried by Na^+^ and K^+^. To model contributions of I_h_ and I_leak_ to ${\left[Na\right]}_{i}$ , we split these currents into Na^+^ and K^+^ components. Concerning the model of I_pump_, every cycle of the pump moves 3 Na^+^ ions out and 2 K^+^ ions into the neuron. The dynamics of ${\left[Na\right]}_{i}$ is governed by Equation 1.


(1) $$\frac{d{\left[Na\right]}_{i}}{dt}=\frac{-\left(\left(\begin{array}{l} {\bar{g}}_{P}m_{P}+{\bar{g}}_{NaF}m_{NaF}^{3}h_{NaF}\\ +{\bar{g}}_{hNa}m_{h}^{2}+g_{\mathrm{leakNa}} \end{array}\right)\left(V_{m}-E_{Na}\right)+3I_{\mathrm{pump}}\right)}{vF}$$

where $E_{Na}$ is the reversal potential for sodium, ${\bar{g}}_{P}$ , ${\bar{g}}_{NaF}$ , and ${\bar{g}}_{hNa}$ are the maximal conductances of I_P_ and I_Na_, and the Na^+^ component of I_h_, correspondingly, and $m_{P}$ , $m_{NaF}$ $h_{NaF}$ , $m_{h}$ are their gating variables, $g_{\mathrm{leakNa}}$ is the conductance of Na^+^ component of I_leak_, $V_{m}$ is the membrane potential, v is the volume of the cell, and *F =* 96,485 C/mol is the Faraday constant. In Equation 1, we used vF = 410 Cnℓ/mol, which gives us realistic size of the neuron: volume 4.2 pℓ and radius 10.05 μm. ${\left[Na\right]}_{i}$ determines the Na^+^ reversal potential: $E_{Na}=\frac{RT}{F}\ln\left(\frac{{\left[Na\right]}_{o}}{{\left[Na\right]}_{i}}\right)$ . ${\left[Na\right]}_{o}$ is constant 0.115 M.

Simulation along the line of coregulation was accomplished by first setting parameter values ( ${\bar{g}}_{h}$ , *I_PumpMax_*) = (0,0.548) and initializing with the standard set of initial conditions (Supplementary Table S1). Then, after simulation, ${\bar{g}}_{h}$ is increased by 0.05 nS, and I_PumpMax_ is decreased in accordance with the coregulation relation established in Ellingson et al. (2021):


(2) $$I_{\mathrm{PumpMax}}\left({\bar{g}}_{h}\right)=0.36+\frac{0.16}{\left({\bar{g}}_{h}+0.85\right)}$$

After each simulation, the values of all state variables were then used as initial conditions in the next simulation. This allowed us to smoothly simulate the action of myomodulin.

### 2.3 Burst analysis

Model activity was analyzed with in-house scripts written in the MATLAB software (The MathWorks, Inc.) in a similar manner as presented in Ellingson et al. (2021).

Depolarized phases of bursting activity were identified as intervals in which the cell rose above-45 mV and remained above this threshold for at least 0.5 s. A burst was defined as an interval in which the cell depolarized and demonstrated continuous spiking activity. Spikes were detected as peaks in the voltage trace above −30 mV, and timing between spikes was used to distinguish full bursts from depolarized phases containing plateau potentials. The beginning of a spike train was identified as a spike which had no predecessor for at least 0.4 s, and the end of a spike train was identified as a spike which had no successor for 0.4 s. If multiple spike trains were detected within a depolarized phase or if no spikes were identified within 0.4 s of the end of a depolarized phase, this depolarized phase would be tagged as a plateau-containing seizure-like oscillation. Depolarized phases which contained an uninterrupted spike-train were identified as bursts. Depolarized phase duration was computed as the interval of time the cell remained above −45 mV. If the depolarized phase was identified as a burst, its burst duration was computed as the interval of time between the first and last spike in the continuous spike train. These measures were nearly identical in bursts (± one interspike interval). Cycle period was computed as the interval between the beginning of a depolarized phase and the beginning of the next depolarized phase. Intraburst spike frequency was computed as the mean of the inverse of interspike intervals within the burst.

In our parameter sweeps of the single HN and HCO models, we observed a variety of different types of activity (Figure 2), which we generally classified as one type of functional activity (functional bursting), or two types of dysfunctional activities: asymmetric bursting, and a plateau-containing activity. Functional bursting was defined as symmetrical or near symmetrical (depolarized phase of neither cell ≥55% of the cycle period) alternation of spiking activity. During a burst, a cell’s membrane potential rises above −45 mV and spikes continuously until it drops below −45 mV. Functional bursting is further differentiated into high-spike-frequency bursting and low-spike-frequency bursting in this article by the intraburst spike frequency (threshold of 18 Hz) and the minimum voltage (threshold of −70 mV) during the post-burst hyperpolarization. Asymmetric bursting activity was defined similarly, but in the asymmetric case bursts of one cell were noticeably longer than those of the other cell. Cases in which the depolarized phase duration of side of the half-center oscillator ≥55% of the period were considered asymmetric. Plateau-like oscillations were characterized by the appearance of plateaus in the depolarized phase of activity, indicating a failure to spike due to a depolarization block.

**Figure 1:**
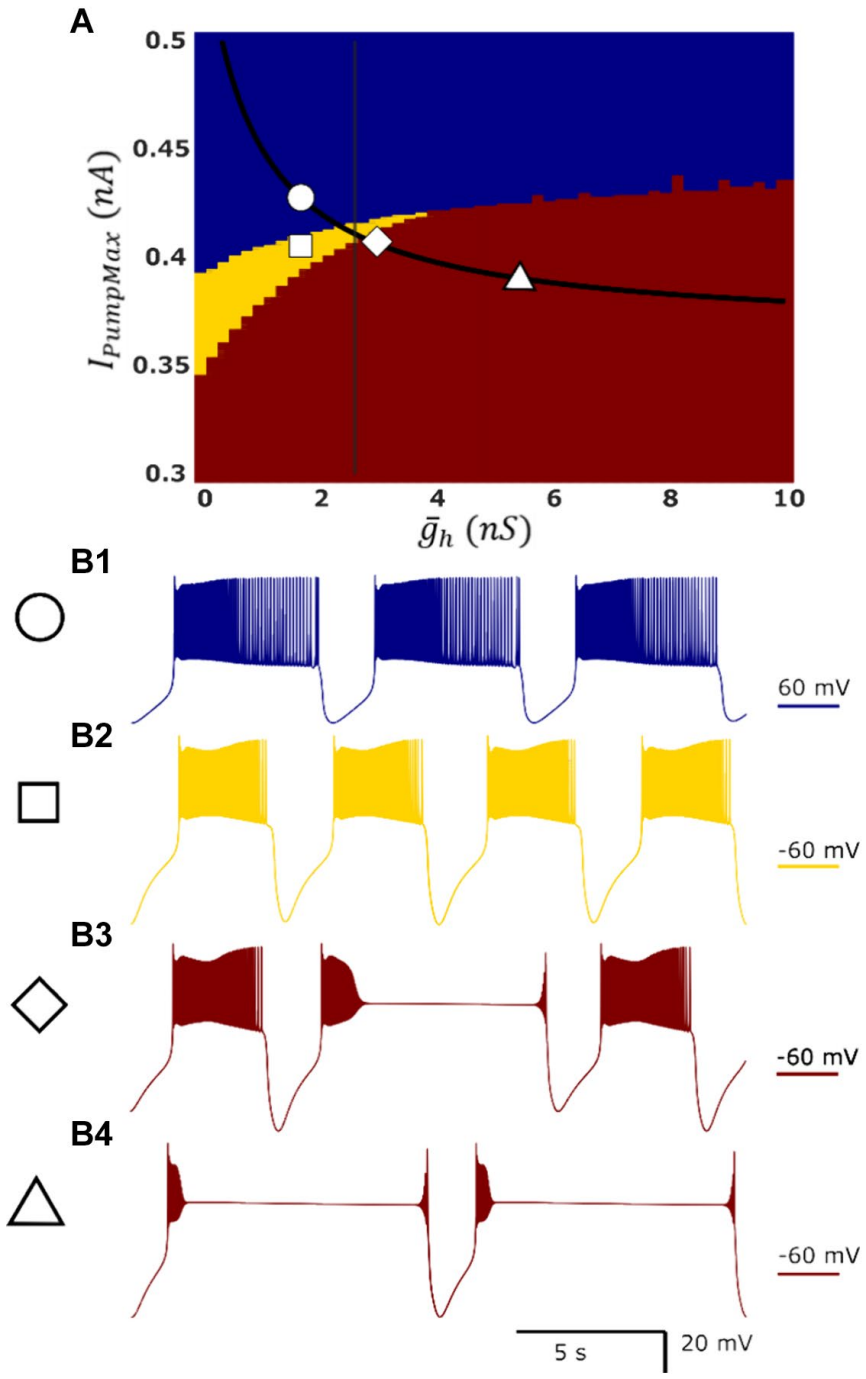
Map of single-cell HN model activity regimes demonstrates the presence of dysfunctional regimes in a parameter domain which is functional in the HCO model. Blue, yellow, and red indicate low-spike-frequency bursting [LSFB, **(B1)**], high-spike-frequency bursting [HSFB, **(B2)**], and plateau-containing oscillations **(B3,B4)**, respectively. Superimposed in black is the myomodulin coregulation path representing experimental data, and the vertical black line denotes the transition into multistability identified in the HCO model along this path. At the control conditions, $({\bar{g}}_{h}\text{,}$ *I_PumpMax_*) = (1.6,0.429), the model displays low-spike-frequency bursting **(B1)**. Reduction in *I_PumpMax_* to $({\bar{g}}_{h}\text{,}$ *I_PumpMax_*) = (1.6,0.4) results in high-spike-frequency bursting **(B2)**. There is a small region of plateau-containing oscillations where high-spike-frequency bursting and plateau regimes appear mixed at the border of the plateau oscillations region **(B3)** $({\bar{g}}_{h}\text{,}$ *I_PumpMax_*) = (2.6,0.406). This border nearly coincides with the entry into bistability in the HCO (vertical black line). The single HN model representing experimental condition Myomodulin 10 uM exhibits only plateau oscillations **(B4)** at $({\bar{g}}_{h}\text{,}$ *I_PumpMax_*) = (5.4,0.385) (triangle), whereas HCO supports two functional bursting regimes (Figures 2B, 3).

Using these definitions of burst and plateau events, regimes of HN activity could be labeled (1) functional low frequency, (2) functional high frequency, (3) asymmetric, or (4) plateau-containing. Single cell activity cannot be asymmetric, so this regime is not considered in analysis of the single cell model.

### 2.4 Spike averaging and phase-wise analysis of currents

A spike averaging algorithm was used to smooth out the spiking contribution to bursts to focus on the average contribution of currents to the underlying oscillatory dynamics of bursting. For each spike, state variables, currents, and fluxes were averaged over its duration in the following manner: Once spike times were identified using previously described methods (Ellingson et al., 2021), spike floors were identified as the minimum value of membrane potential between spikes. These points were then used to compute spike duration and to smooth time series of state variables, currents, and Na^+^ fluxes. Between spike floors, all values for a trace were set to the average value between spike floors. To compute average current and flux contributions over a cycle, smoothed depolarized and hyperpolarized phases were sliced out of a cycle, and traces were integrated using the trapezoidal Riemann sum method (function *trapz* in MATLAB) and normalized to the duration of the phase for comparison at different cycle periods.

## 3 Results

We developed a model of a leech heart interneuron half-center oscillator (HCO) and described a neuromodulation-governed mechanism for robust control of cycle period (Ellingson et al., 2021). We also identified specific pairs of model parameter values for $I_{\mathrm{PumpMax}}$ and ${\bar{g}}_{h}$ that represent experimentally recorded activities in HCOs under variation of myomodulin concentration. We captured the effects of myomodulin by a simple coregulation path. This was achieved by curve-fitting the identified parameter pairs to an inverse relationship (Ellingson et al., 2021) expressed by Equation 2.

Based on our modeling analysis, we demonstrated that by following this myomodulin coregulation path, the HCO avoids a dysfunctional asymmetric bursting regime at higher levels of $I_{\mathrm{PumpMax}}$ and ${\bar{g}}_{h}$ as well as a dysfunctional seizure-like plateau-containing regime at lower levels of $I_{\mathrm{PumpMax}}$ and in doing so achieves larger ranges of burst duration and bursting cycle period.

### 3.1 Co-existence of low- and high-spike frequency bursting regimes in the HCO model

**Figure 2:**
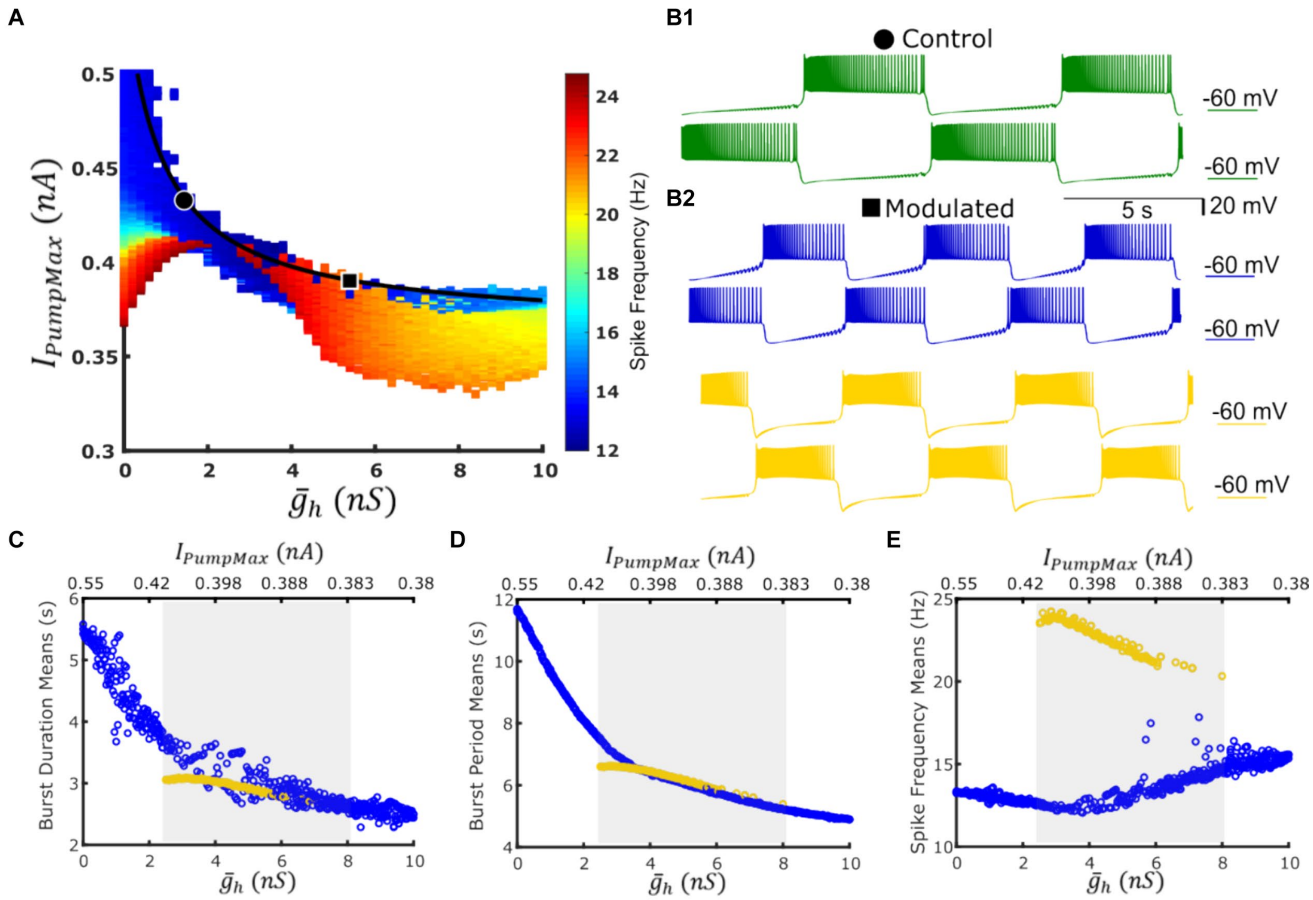
A coregulation path traverses a domain of co-existent low-spike-frequency and high-spike-frequency bursting regimes with roughly the same cycle period while navigating between dysfunctional regimes. **(A)** Map of spike frequency in the functional regime overlaid with the experimentally validated path of coregulation in black; dysfunctional regimes are marked in white; points representing control and Myomodulin 10 uM conditions are marked by a black circle or square, respectively. **(B1)** Bursting activity under control conditions (green trace) is obtained at $({\bar{g}}_{h}\text{,}$ *I_PumpMax_*) = (1.6,0.429). **(B2)** Two functional bursting regimes co-exist at the parameter coordinates $({\bar{g}}_{h}\text{,}$ *I_PumpMax_*) = (5.4,0.385) corresponding to experimental Myomodulin 10 uM conditions: low-spike-frequency bursting (blue traces on the top) and the high-spike-frequency bursting (yellow traces on the bottom). Burst duration **(C)**, burst period **(D)**, and spike frequency **(E)** from simulations along the path of coregulation. Blue points represent the low-spike-frequency regime, and yellow points represent the high-spike-frequency regime, the shaded gray area indicates the multistable domain of parameters, where either regime can manifest given appropriate initial conditions or targeted perturbations.

A two-parameter map of spike frequency indicated emergence of a high-spike-frequency regime within the boundaries of the functional activity of the HCO model (Figure 2A). It’s typical mean intra-burst spike frequency (i.e., spike frequency) was roughly two times higher compared with the low-frequency regime; 24 Hz versus 12 Hz, respectively. Given the sharp transition from the low-spike-frequency bursting (LSFB) regime to the high frequency bursting (HSFB) regime and occasional appearance of the low frequency regime within the domain of the high frequency regime on the map, we hypothesized that these two regimes may coexist within some domain along the experimentally-substantiated myomodulin coregulation curve. This growth of the spike frequency was qualitatively consistent with the trend recorded experimentally (Tobin and Calabrese, 2005) that spike frequency increases under the application of myomodulin by 10–30%.

At the parameter pair ( ${\bar{g}}_{h}$ , *I_PumpMax_*) = (1.6, 0.429), which represented activity observed in control experiments, model activity is monostable (Figure 2B1). At the parameter pair ( ${\bar{g}}_{h}$ , *I_PumpMax_*) = (5.4,0.385), which corresponded to application of 10 uM myomodulin, the HCO can produce either the low-spike-frequency regime or the high-spike-frequency (Figure 2B2) depending on the set of initial conditions. To test for multistability along the curve of coregulation, we performed continuous simulation along the path of coregulation by incrementing ${\bar{g}}_{h}$ and decrementing *I_PumpMax_* in accordance with the coregulation relation Equation 2 with three methods of initializing model state variables (Methods). We noticed the appearance of the high-spike-frequency regime at ( ${\bar{g}}_{h}$ , *I_PumpMax_*) = (2.5,0.408). Along with the myomodulin coregulation path toward high myomodulin concentration, the HCO system is capable of producing this high-spike-frequency regime until ( ${\bar{g}}_{h}$ , *I_PumpMax_*) = (8, 0.378), after which the system becomes monostable on the low-frequency regime once again. When the initial conditions were appropriately chosen within the specified parameter boundaries, the HCO neurons were able to exhibit either the low-frequency regime or the high-frequency regime. This was confirmed by simulation for a duration of at least 1,600 s of model time. In both regimes, we observed a decrease in bursting cycle period and burst duration at very similar values as modulatory coregulation proceeds to higher ${\bar{g}}_{h}$ and lower *I_PumpMax_* (Figures 2C,D). However, the high-spike-frequency regime decreases in spike frequency from 24 Hz to 20 Hz as the coregulation proceeds, while the low spike frequency regime increases in spike frequency from 12 Hz to 15 Hz throughout the domain of bistability (Figure 2E). Thus, along the curve of coregulation, there is a region of multistability in the HCO model between ( ${\bar{g}}_{h}$ , *I_PumpMax_*) = (2.5,0.408) and ( ${\bar{g}}_{h}$ , *I_PumpMax_*) = (8, 0.378) in which the typical bursting, e.g., LSFB, coexists with the HSFB regime.

### 3.2 Regime switching

While both regimes are consistent with functional activity as measured in experiments with HCOs (Kueh et al., 2016; Wenning et al., 2018; Erazo-Toscano et al., 2023), a major question arises as to how the nervous system can control transitions between regimes in order to utilize the potential benefits of the high-spike frequency bursting regime. To investigate this, we mimicked the effect of post-synaptic currents (PSCs) originating from outside the HCO – perhaps as a descending central command or as an input from elsewhere within the full circuit of the leech heartbeat central pattern generator. We represented excitatory and inhibitory PSCs by pulses of conductance for a current with corresponding reversal potential. We were able to produce switches from LSFB to HSFB and vice versa with either excitatory or inhibitory pulses. We found that the success of the switch depended on when within a burst cycle the pulse was delivered and on the magnitude of the conductance pulse.

To investigate the ability to switch between these co-existing regimes we chose a point on the coregulation path at ( ${\bar{g}}_{h}$ , *I_PumpMax_*) = (5.4,0.385), representing experimental data with 10 μM Myomodulin applied (Ellingson et al., 2021). Simulation of LSFB and HSFB were accomplished by setting parameter values (${\bar{g}}_{h}$*, I_PumpMax_*) = (5.4,0.385) and initializing with LSFB and HSFB initial conditions, respectively (Supplementary Table 2). The cycle periods for low-and high-spike frequency bursting were notably close, with the former being 5.769 s and the latter slightly longer at 6.184 s. For LSFB, the burst durations of the HCO neurons V_1_ and V_2_ were 2.42 s and 3.01 s, respectively, exhibiting an asymmetry in the pattern (Assym = 0.22), evaluated following (Ellingson et al., 2021). Conversely, for HSFB, the burst durations for V_1_ and V_2_ were both 2.75 s, showing no pattern asymmetry (Assym = 0).

**Figure 3:**
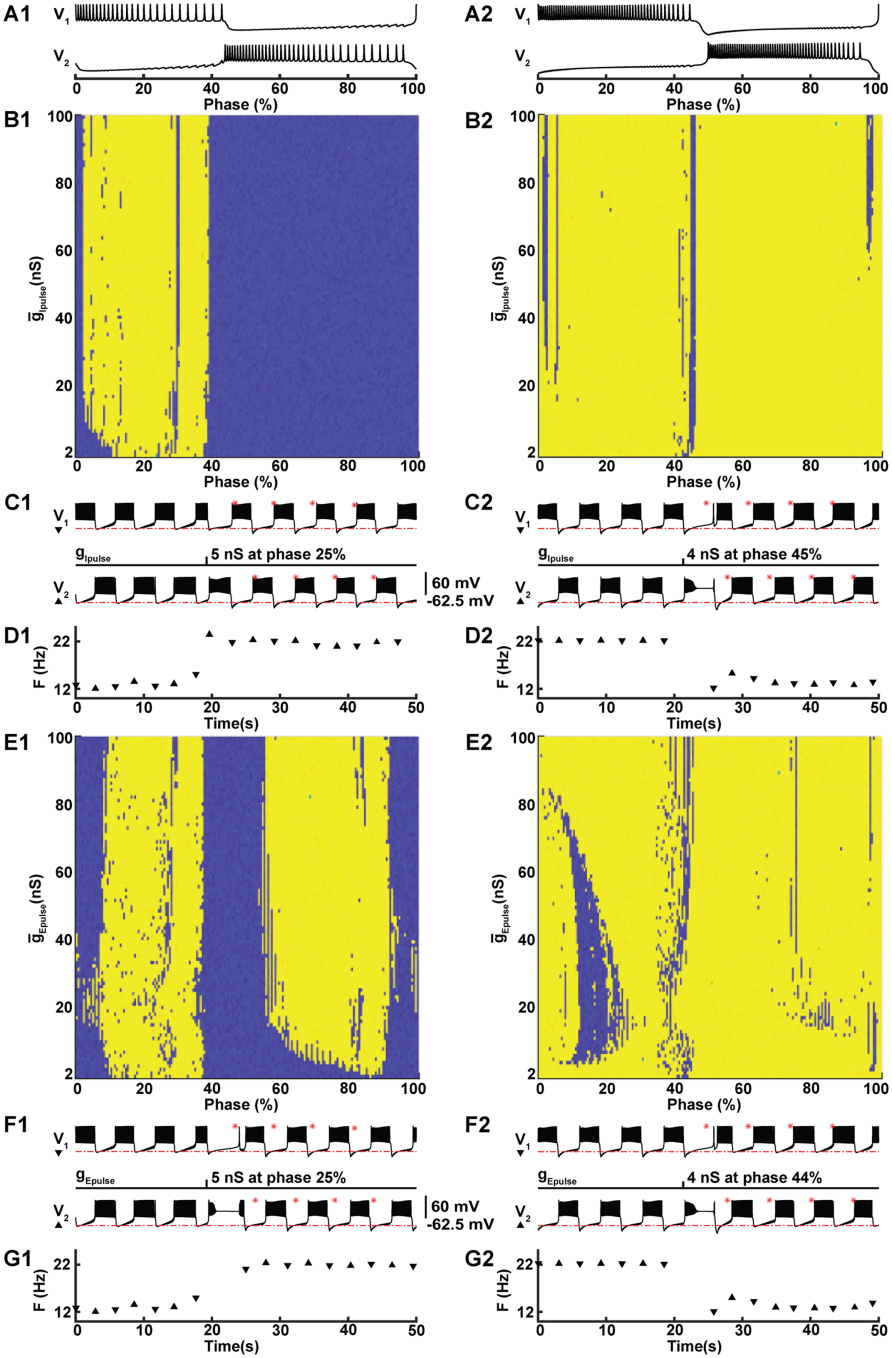
Under modulation, perturbation with either an excitatory or an inhibitory conductance pulse can trigger a switch between the two coexisting HCO activity regimes. At $({\bar{g}}_{h}\text{,}$ *I_PumpMax_*) = (5.4,0.385), the HCO model can be switched from the low-spike-frequency bursting (LSFB, blue) to the high-spike-frequency bursting (HSFB, yellow) and vice versa by a 30 ms inhibitory **(B1,B2,C1,C2,D1,D2)** or an excitatory **(E1,E2,F1,F2,G1,G2)** pulse of conductance with reversal potentials −0.0625 V and 0.0 V, respectively. The pulse was applied to one neuron, marked as V_1_. The success of the switch depends on the phase **(A1,A2)**, starting with the first spike of a burst of V_1_, and the amplitude of the pulse conductance **(B1,B2,E1,E2)**. Examples of switches from LSFB to HSFB **(C1)** and vice versa **(C2)** with an inhibitory pulse. **F1** and **F2** exhibit similar examples of switches triggered by excitatory pulses. Panels **D1**,**D2**,**G1**,**G2** exhibit the transitions across the threshold frequency 18 Hz.

By delivering individual pulses with varying magnitudes at different phases of the burst cycle, we investigated the properties of the pulses that triggered a switch. We applied 30 ms pulses of conductance with either an inhibitory (−62.5 mV) or an excitatory (0 mV) reversal potential to one neuron in our HCO model (Figure 3). Since the HCO model is symmetric, for the sake of clarity, we delivered the pulse to a neuron shown at the top, referred to as V_1_. Marking the beginning of a cycle with the first spike in the burst of this neuron, we defined the phase of the pulse delivery as the moment in time within a burst cycle, which we normalized by dividing it by the cycle’s duration, and expressed it as a percentage (Figures 3A1,A2). We methodically varied the conductance of these pulses in a range from 1 nS to 101 nS, increasing the conductance in increments of 1 nS for each subsequent pulse. We also delivered pulses at different phases. For HSFB, we applied pulses at 200 different phases, with each phase separated by a time interval of 0.03092 s. We employed the same time interval for LSFB, which resulted in a total of 189 phases, note that the cycle period for LSFB is shorter compared to HSFB. Sweeping systematically pulse amplitude and the phase we mapped 20,000 and 18,900 points for HSFB and LSFB perturbations, respectively. To test whether the switch occurred, we established a threshold frequency of 18 Hz to distinguish between LSFB and HSFB. Counting the switch cases on the maps (Figures 3B1,B2,E1,E2), we found that it is much easier to switch from LSFB to HSFB than vice versa by either an inhibitory or an excitatory pulse with success in 33.7 and 59.5% cases, respectively. Accordingly, switches from HSFB to LSFB were less common. However, our findings indicate that excitatory and inhibitory pulses still facilitated these switches in 4.1 and 9.9% of cases, respectively. This data implies that using an excitatory pulse is more than twice as effective in inducing a switch compared to an inhibitory pulse.

### 3.3 Single-cell HN model under modulation

When surgically or pharmacologically isolated and thus without the synaptic input from the rest of the CPG, HN cells can burst endogenously (Cymbalyuk et al., 2002; Erazo-Toscano et al., 2023). Likewise, our single HN model bursts under control conditions, ( ${\bar{g}}_{h}$ , *I_PumpMax_*) = (1.6,0.429). Based on the prevalence of seizure-like plateau-containing oscillations at low values of *I_PumpMax_* in the HCO model (Ellingson et al., 2021), we hypothesized that the mutually inhibitory interactions of the HCO may be preventing a depolarization block in at least some of the parameter regions of the functional HCO bursting regime. The high-spike frequency regime seen in the HCO model at lower levels of *I_PumpMax_* could be an intermediate regime between low-spike frequency activity and a depolarization block leading to plateaus under higher levels of modulation. To test this hypothesis, we first performed a two-dimensional sweep of $I_{\mathrm{PumpMax}}$ and ${\bar{g}}_{h}$ on the single-cell HN model, investigating the same modulatory parameter domain as in our HCO simulations.

**Figure 4:**
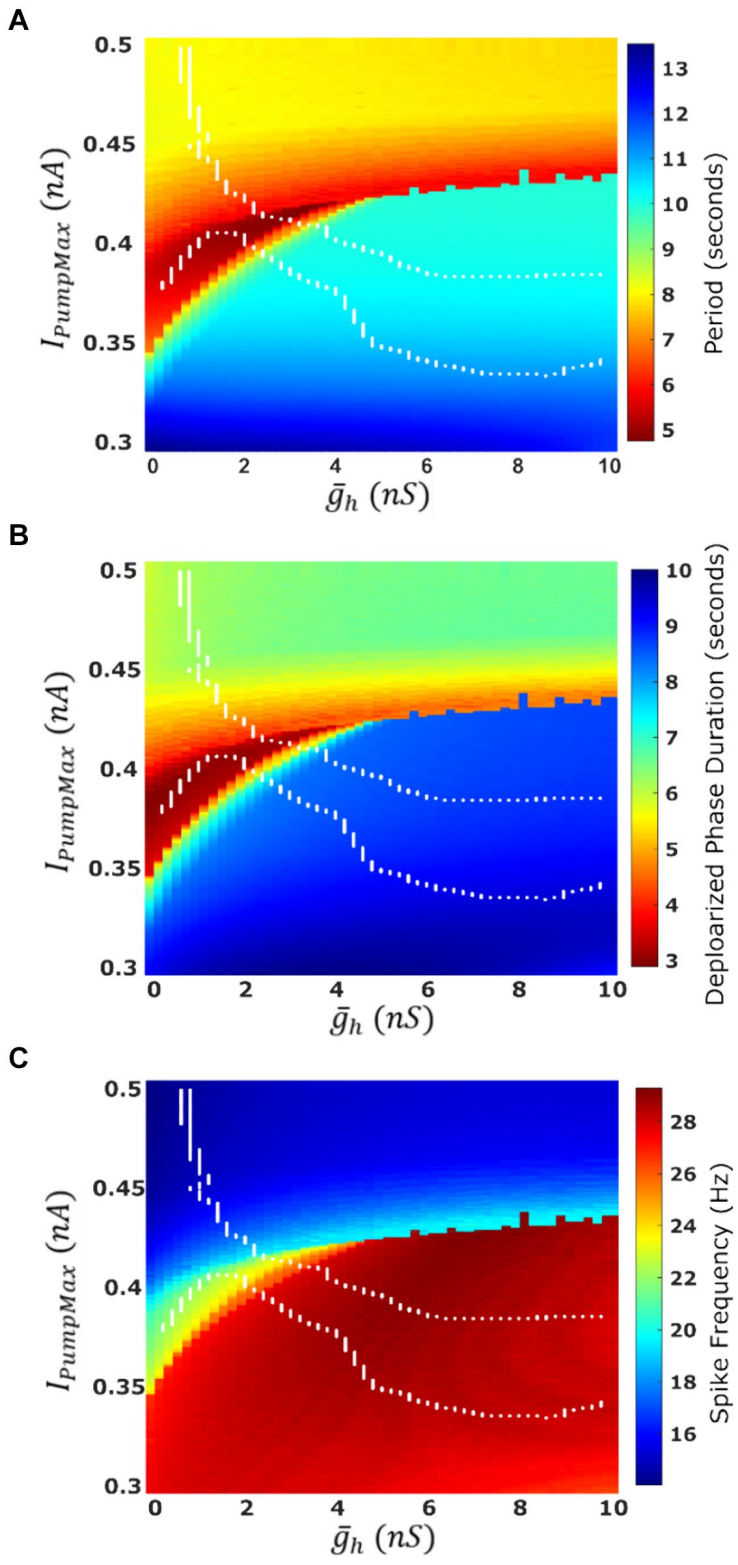
Characteristics of the oscillatory activities of a single-cell HN model under two parameter variation $({\bar{g}}_{h}\text{,}$ *I_PumpMax_*). **(A)** Average cycle period of single cell activity. Warmer colors indicate a faster rhythm. Cycle period is more sensitive to parameter *I_PumpMax_* than to ${\bar{g}}_{h}$ . **(B)** Average duration of the depolarized phase of bursting or plateau-containing oscillations. **(C)** Average spike frequency within the depolarized phase. The boundaries of the HCO functional activity regime are superimposed in white.

The most apparent difference between oscillatory activity produced by a decoupled HN neuron versus being incorporated in an HCO was in the control of cycle period. In the HCO system, the gradient of period was most closely associated with changes in ${\bar{g}}_{h}$ (Ellingson et al., 2021). In the single cell, however, throughout most of the tested parameter domain, changes in ${\bar{g}}_{h}$ have little effect; $I_{\mathrm{PumpMax}}$ plays a much bigger role in regulation of the period (Figure 4A). As a demonstration, consider any arbitrary vertical path through the parameter domain shown in Figure 4A. At high levels of $I_{\mathrm{PumpMax}}$ , cycle period is around 8 s, and drops as low as 5 s as $I_{\mathrm{PumpMax}}$ is reduced. At middling values of $I_{\mathrm{PumpMax}}$ this trend abruptly reverses. Cycle period jumps to nearly 10 s and increases to as much as 13.5 s as $I_{\mathrm{PumpMax}}$ is continuously reduced. The parameter ${\bar{g}}_{h}$ appears to play a role in determining the level of $I_{\mathrm{PumpMax}}$ which will induce this trend reversal. At ${\bar{g}}_{h}$ = 0 nS, this reversal occurs at *I_PumpMax_* = 0.35 nA, but at ${\bar{g}}_{h}$ = 10 nS, this reversal occurs at *I_PumpMax_* = 0.435 nA. This border also appears in maps of depolarized phase duration (Figure 4B) and spike frequency (Figure 4C) suggesting that the single cell undergoes at least one major regime change under modulation. Using our previous methods for identifying plateau potentials in membrane activity (Ellingson et al., 2021), we found that beyond this border at which the period trend reverses, 100% of membrane potential oscillations contain plateau potentials (Figure 1).

### 3.4 Single cell HN model displays several regimes of activity

Similar to the HCO model, based on the above burst characteristics, we were able to categorize single cell activity into three types of regimes: low-spike-frequency bursting activity, high-spike-frequency bursting activity, and seizure-like plateau-containing activity (Figure 1, blue, yellow, and red, respectively). Between the regions of high-spike-frequency bursting and plateaus, we also identified a small domain of hybrid regimes consisting of high-spike-frequency bursting and plateaus (Figure 1B3). The high-spike-frequency regime is qualitatively very similar to the high-spike-frequency regime identified in the HCO model, in the same way that the low-spike-frequency regime is qualitatively similar to the low-spike-frequency regime in the HCO model. Without synaptic inhibition, the HN neurons are able to recover more quickly to initiate the next burst leading to a shorter interburst interval.

We mapped the experimentally derived HCO domain of functional bursting (Figure 2A) with two white dashed curves, marking borders (Figure 4A), onto the corresponding parameter space of a single HN activities (Figure 4A). Then comparing this map to the established map of the activity regimes (Figure 1A), we noticed that the transition from bursting to plateau-like oscillations in the HN model along the coregulation path occurs at ( ${\bar{g}}_{h}$ , *I_PumpMax_*) = (2.6,0.406). Mutual synaptic inhibition, at middling to high levels of ${\bar{g}}_{h}$ is sufficient to prevent this depolarization block, allowing for HCO functional activity in parameter spaces which would not be functional without the synaptic inhibition. In fact, 67% of parameter pairs identified as functional in the HCO (1,532 points out of 2,278, Figure 2A), are dysfunctional in a single cell (Figures 1A, 4A). The point at which the single cell transitions to seizure-like plateau-containing activity coincides with the beginning of the domain of multistability in the HCO map. At the same level of modulation, which transitions the single cell into plateaus, the HCO still maintains functional activity, and also gains coexistence of low and high-spike-frequency regimes. In addition, when *I_PumpMax_* is decreased from control values in the single HN, the cell transitions to the high-spike-frequency bursting regime at low levels of ${\bar{g}}_{h}$ .

### 3.5 Description of regimes and ionic mechanisms

**Figure 5:**
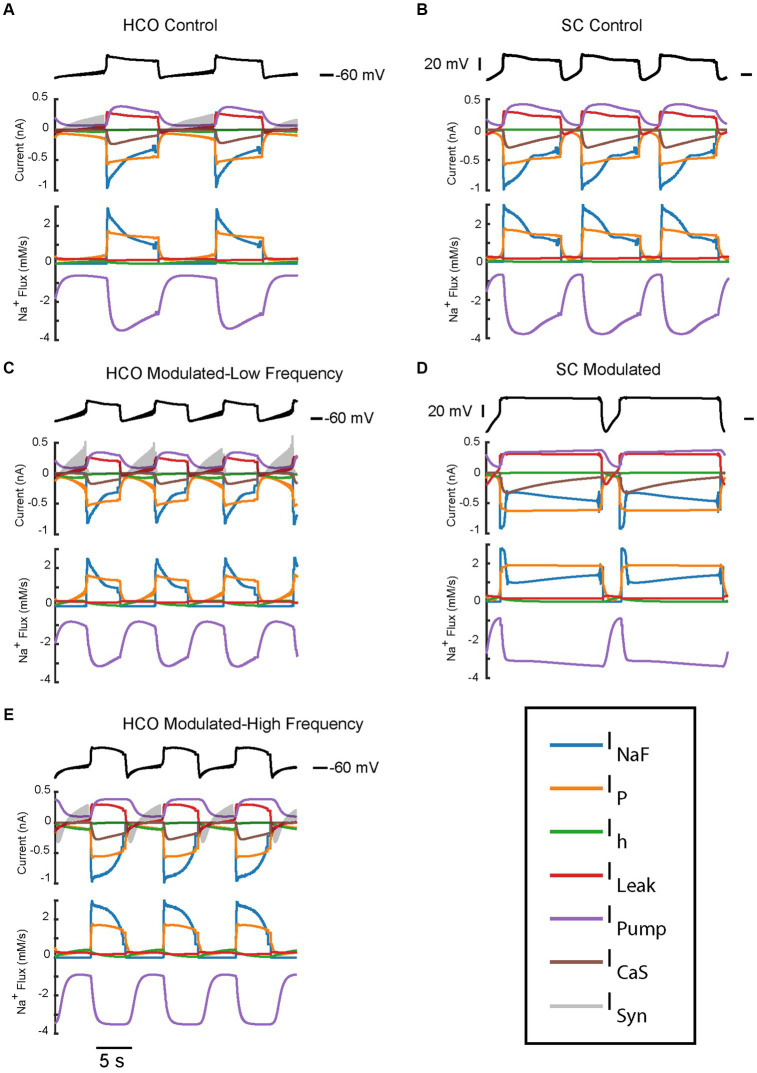
Current and Na^+^ flux time series of the two bursting regimes produced by the HCO and single HN models under control and modulated conditions. All traces have been averaged over spikes to provide a look at the oscillatory dynamics. Current and Na^+^ flux under control parameters $({\bar{g}}_{h}\text{,}$ *I_PumpMax_*) = (1.6,0.429) for HCO **(A)** and single HN **(B)**, both of which are low frequency bursting regimes. Current and Na^+^ flux under modulated parameters $({\bar{g}}_{h}\text{,}$ *I_PumpMax_*) = (5.4,0.385) for HCO, which is bistable – capable of producing either a low-spike-frequency **(C)** regime or a high-spike-frequency regime **(E)** and single HN **(D)** which is a plateau oscillations regime.

**Figure 6:**
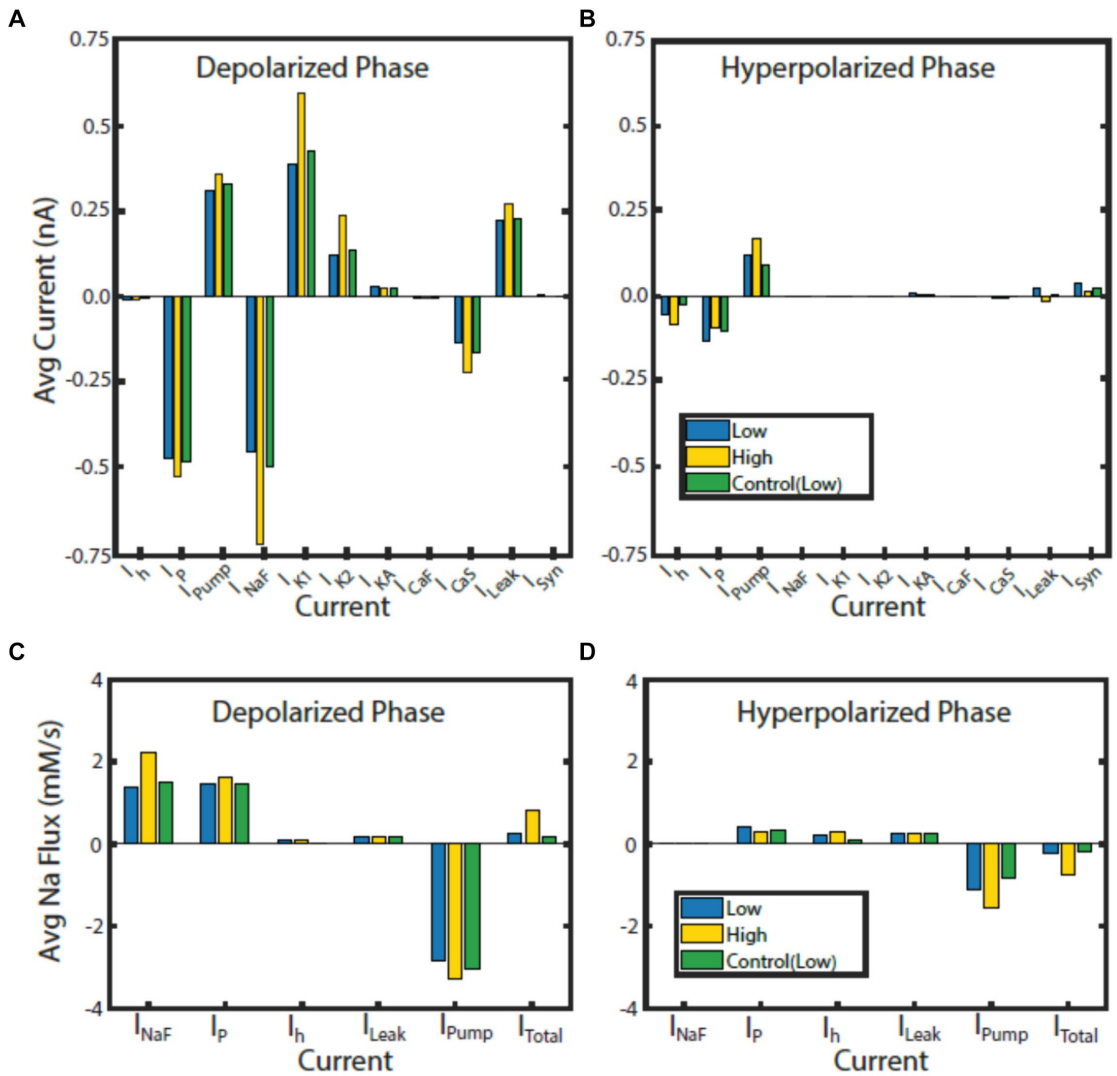
Contributions of currents to bursting phases normalized to phase duration in HCO model regimes. Average current for the monostable control (green) regime at $({\bar{g}}_{h}\text{,}$ *I_PumpMax_*) = (1.6, 0.429), and the high (gold) and low (blue) spike-frequency regimes at bistable point $({\bar{g}}_{h}\text{,}$ *I_PumpMax_*) = (5.4,0.385) during the depolarized **(A)** and hyperpolarized phase **(B)**. Average Na^+^ flux for each Na^+^ carrying current for the control (green) regime at $({\bar{g}}_{h}\text{,}$ *I_PumpMax_*) = (1.6, 0.429), high (gold) and low (blue) spike-frequency regimes at bistable point $({\bar{g}}_{h}\text{,}$ *I_PumpMax_*) = (5.4,0.385) during the depolarized **(C)** and hyperpolarized phase **(D)**.

**Figure 7:**
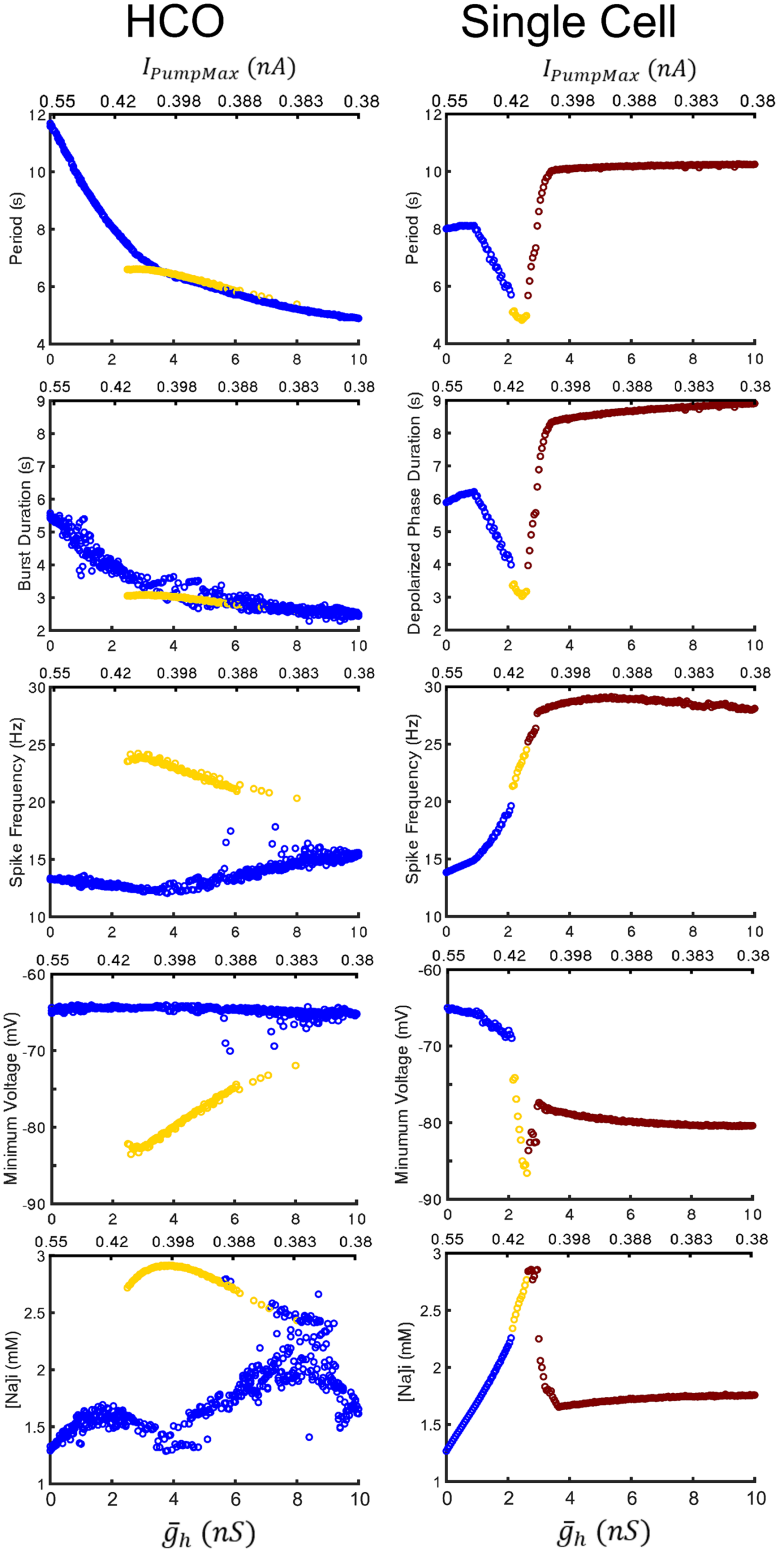
Comparison of characteristics of the HCO and single HN model oscillatory activities under two parameter variation along the coregulation path. The low-spike-frequency bursting, the high-spike-frequency bursting, and plateau-containing oscillations are marked in blue, yellow, and red-brown, respectively. The HCO model exhibits functional bursting over the whole range of parameter variation with intra-burst spike frequency, minimal membrane potential, and amplitude of the intracellular Na^+^ concentration oscillations being notably distinct. In the single HN model, the low-spike-frequency and high-spike-frequency bursting share a trend and transition into pathological seizure-like plateau oscillations in all presented characteristics. Upper left three panels repeat Figures 2C–E for comparison purposes.

In this section, we compare trajectories and contributions of the key ionic currents supporting the bursting regimes of the HCO and single cell (Figures 5–7). To investigate the roles of different currents in the underlying dynamics of large oscillations of the membrane potential which include bursts and plateaus, we applied a spike averaging algorithm to the depolarized phase of model voltage traces and currents. This smooths out the contribution of individual spikes to highlight the underlying oscillations which bring the cell to spiking threshold. As can be seen from Figure 7, a notable difference in the characteristics of the low-spike-frequency regime (blue) and the high-spike-frequency regime (yellow) is that the amplitude of intracellular Na^+^ concentration oscillations, which controls the level of pump current, is larger in the high-spike-frequency one. Remarkably, in the HCO it is nearly twice as high in the high-spike-frequency regimes as in the low spike frequency regimes (Figure 7). This results in a higher pump current throughout the burst as well. In low-spike-frequency regimes, the pump current rises and falls during the burst (Figure 5). In high-spike-frequency and plateau-containing regimes, the pump current rises monotonically during the depolarized phase and remains high until the minimum membrane potential is reached in the hyperpolarized phase. This suggests that pump current plays important role in the dynamics of burst termination in these regimes. The pump current remains high in these regimes at the burst termination adding momentum to the outward currents which hyperpolarize the membrane resulting in a much larger afterhyperpolarization. This in turn pushes the membrane potential far below the synaptic reversal potential (−62.5 mV). This means that at the beginning of the hyperpolarized phase, the synaptic current is depolarizing during some time interval, despite it normally functioning as inhibitory.

Since the intracellular Na^+^ concentration governs the pump current and is in turn affected by the pump current, we assessed the average contribution of currents to Na^+^ flux during the depolarized and hyperpolarized phases of activity for the HCO regimes of interest (Figure 6). Analysis of individual currents shows that I_NaF_ and I_P_ are the major drivers of depolarization and intracellular Na^+^ concentration in all regimes of activity. The higher spike-frequency in the high-spike-frequency regime compared to the low-spike-frequency regime brings in much more Na^+^ through I_NaF_ in the depolarized phase. Higher levels of intracellular Na^+^ increase pump current activation, so the pump is more active and contributes more heavily to the removal of Na^+^.

In the HCO high-spike-frequency regime, the majority of currents have a higher average magnitude during both the depolarized and hyperpolarized phases when compared to low-spike-frequency regime. The high-spike-frequency regime does not appear above an *I_PumpMax_* of 0.42 nA, but the low-spike-frequency regime does appear at high maximal pump currents (i.e., at control conditions). Yet despite only appearing at lower values of *I_PumpMax_*, the HCO high-spike-frequency regime has a higher average pump current throughout the burst than in the control regime (Figure 7). Through coregulation, a reduction in *I_PumpMax_* of 10% from control leads to an 8% increase in average pump current over the burst when the HCO transitions into the high-spike-frequency regime. There are several major exceptions, however, to this idea of higher total current in high-spike-frequency regimes. Persistent Na^+^ current, I_P_, is smaller during the hyperpolarized phase of the high-spike-frequency regime than the low spike-frequency regime. Leak current is an inward current on average during the hyperpolarized phase of the high-spike-frequency regime, largely due to the massive afterhyperpolarization associated with this regime. Synaptic current, for the same reasons, appears to be smaller on average during the hyperpolarized phase of the high-spike-frequency regime. In the high-spike-frequency regime, the afterhyperpolarization is great enough to cross the reversal potentials for the leak and synaptic currents and thus briefly reverses their contributions to the current balance on average.

In all regimes, the pump current is the sole source of the outward Na^+^ flux, and so is unsurprisingly critical in maintaining the Na^+^ electrochemical gradient. The major inward contributors of Na^+^ flux during the depolarized phase are I_NaF_ and I_P_, while the major contributors during the hyperpolarized phase are I_P_, I_h_, and I_Leak_. What accounts for the major difference in the amplitude of Na^+^ oscillations between low and high-spike-frequency regimes is principally the I_NaF_ Na^+^ entry driven by the higher spike frequency.

We next performed a continuous simulation changing parameters ${\bar{g}}_{h}$ and *I_PumpMax_* along the line of coregulation to assess the effects of modulation on a single HN cell (Figure 7) directly. We found that the cell transitions between the low-spike-frequency and high-spike-frequency regimes at ( ${\bar{g}}_{h}$ , *I_PumpMax_*) = (2.15,0.413), and to the hybrid plateau containing regime at ( ${\bar{g}}_{h}$ , *I_PumpMax_*) = (2.6,0.406), and then to full plateaus at ( ${\bar{g}}_{h}$ , *I_PumpMax_*) = (3.0,0.402). The entrance into the domain of multistability by the HCO model approximately coincides with the transition of the single cell into the area producing hybrid plateau-containing activity like that shown in Figure 1B3. Under increasing levels of modulation in the single HN model, period and depolarized phase duration decreased until the transition into plateaus. The HCO model, however, continues to monotonically decrease in period and depolarized phase duration throughout the entire tested modulatory parameter space. Single HN spike frequency increases monotonically through both the low and high-spike-frequency regimes, suggesting that there could be a smooth transition between regimes in this case. This is unlike the HCO model, in which the transition into the high spike-frequency regime is abrupt, and then displays a decrease in spike frequency under progressively higher levels of modulation, i.e., higher values of ${\bar{g}}_{h}$ and lower *I_PumpMax_*. Yet even in the single cell, the apparent trends of minimum membrane potential in the hyperpolarized interval are different between the low-spike-frequency and high-spike-frequency regimes. This suggests that the two regimes are actually operating using distinct bursting mechanisms, and this is even clearer in the HCO model, in which minimum membrane potential is constant around −65 mV for the low spike-frequency regime, but the minimum membrane potential of the high-spike frequency regime is much lower, and trends upward through the bistable domain from −85 mV to −73 mV under higher levels of modulation. The measured burst characteristics which most clearly differentiate the two regimes are the spike frequency, the minimum membrane potential in the hyperpolarized interval, and the amplitude of [Na]_i_ oscillations. Thus, results presented in Figure 7 confirms that the major distinctions between the regimes are observed over the ranges of the coregulated parameters.

## 4 Discussion

Neuromodulation of rhythmic circuits adjusts their patterns of activity to environmental challenges or altering behavioral goals by arranging orchestrated changes of ionic currents. Previously we have shown that application of endogenous leech neuromodulator myomodulin to leech heartbeat half-center oscillators coordinates a decrease of pump current and an increase of h-current and thus adjusts the alternating bursting pattern in a wide range of the cycle period and burst duration (Masino and Calabrese, 2002; Tobin and Calabrese, 2005). In a biophysical model, this coregulation of two currents expands the functional range of the governed biophysical parameters, maximal pump activity and maximal conductance of h-current (Ellingson et al., 2021). Here, we demonstrate that this myomodulin coregulation leads to emergence of a new bursting regime with high spike frequency and roughly the same cycle period. This regime co-exists with a low spike frequency bursting regime along the coregulation of the two currents. Comparing the activity regimes of the HCO and single HN models, we show that neuromodulation induces plateau-containing seizure-like oscillations in the isolated HN neuron. The point of the breakdown into this dysfunctional activity is very close to the point at which modulation gives rise to the coexistent regimes in the HCO model. Thus, the synaptic inhibition harnesses the seizure-like regime into the two functional bursting regimes with disparate spike frequencies. The new high-spike-frequency bursting regime provides an opportunity for a higher level of synaptic input to motor neurons during modulation. Counterintuitively, in the higher spike frequency regime, lowering $I_{\mathrm{PumpMax}}$ by neuromodulation leads to an increase of the pump generated outward current.

## 5 Neuromodulation can cause multi-stability of activity regimes

Changes of the biophysical properties that govern neuronal dynamics can result in the co-existence of activity regimes near the transitions between these regimes (Bos et al., 2021). The coexistence is discernible through the identification of state-dependent activity, the observation of hysteresis associated with transitions between different regimes, and the ability to trigger a lasting or permanent switch between the regimes using short pulse perturbations. A number of neuronal models have been shown to exhibit the coexistence of different activity regimes, including silence and spiking, silence and bursting, spiking and bursting, as well as the coexistence of bursting regimes with distinct properties (Lechner et al., 1996; Newman and Butera, 2010; Malashchenko et al., 2011a,b; Marin et al., 2013).

Consistently, neuromodulation-induced changes to neurons can lead to state-dependent transitions (Doi and Ramirez, 2010; Marder et al., 2014; Sharples and Whelan, 2017) and multistability (Hounsgaard and Kiehn, 1989; Lechner et al., 1996). One prominent example is the induction of multistability in neuron R15 within the abdominal ganglia of *Aplysia* by serotonin (5-HT) (Canavier et al., 1993, 1994; Butera et al., 1995; Butera, 1998). In a biophysical model of R15, under the effects of modeled 5-HT application, the neuron exhibits multistability, producing either co-existing bursting and beating patterns or multiple co-existing bursting patterns, yet under control conditions this neuron exhibits only one bursting regime (Butera et al., 1995). Notably, up to seven bursting regimes were reported to co-exist in the model under the influence of neuromodulation. Model predictions were corroborated in follow-up experiments demonstrating the co-existence of beating and bursting regimes (Lechner et al., 1996) and a series of co-existing bursting regimes differing in the number of spikes by one (Newman and Butera, 2010). The functional role of this multistability in R15 is not well understood.

### 5.1 Merits of multistability

To generate various rhythmic patterns, a central pattern generator circuit can implement two conceptually different mechanisms: either it moves through a reversible smooth transition between patterns or through state-dependent abrupt transitions exhibiting hysteresis. The latter implies the coexistence of neighboring regimes, enabling controlled switching between them. Central pattern generators in both vertebrates and invertebrates can employ either of these mechanisms, exhibiting gradual changes of the speed of their patterns or transitioning between rhythms characterized by orders-of-magnitude differences in time scales. Examples include in vertebrates dopamine-induced continuous and episodic locomotor bursting regimes of postnatal mouse spinal cord preparation (Sharples and Whelan, 2017; Sharples et al., 2021), intact cat’s paw-shaking and locomotion (Parker et al., 2021), as well as various mammalian breathing patterns (Lieske et al., 2000), and in invertebrates, swimming and crawling behaviors of medicinal leeches and sea slugs (Popescu and Frost, 2002; Briggman and Kristan, 2006), fast and slow swimming of jellyfish (Mackie and Meech, 1985), two distinct gastric mill patterns of the crab stomatogastric nervous system that are induced either by ventral cardiac neurons or by postoesophageal commissure neurons (White and Nusbaum, 2011). These CPG alternative configurations either share a core circuit or share some subset of circuitry and engage other circuit modules in the process of modulation toward transition (Briggman and Kristan, 2008). It is harder to distinguish these basic mechanisms in the vertebrate preparations with larger numbers of neurons. Recently, in zebrafish preparation, a modular organization has been established where the speed of locomotion increases through sequential engagement of corresponding-to-speed modules along with the increase of neuromodulation (Pallucchi et al., 2024).

It is notable that in a CPG with multiple co-existing functional rhythms, no ongoing or permanent changes to the network or the neurons would be required to switch a pattern. A single pulse of conductance representing a single post-synaptic potential can be sufficient to change the activity pattern or induce a transient response on a smaller time-scale like locomotion and paw-shaking in cats (Parker et al., 2018, 2021). The ability to switch between functional patterns with a single pulse is energy efficient, although it requires the neuronal system to be already dynamically capable of producing multiple regimes. With the type of multistability identified here, with the smaller distributed heartbeat CPG (assembled of bilateral pairs of HN cells), our results suggest that a rhythmic neural system could increase spike frequency without changing the underlying cycle period of bursting merely by a single pulse of post-synaptic current. This transition would allow for a more effective synaptic input to motor neurons or muscles controlled by a CPG while maintaining the functional rhythm. Such a regime could exploit muscle dynamics for more efficient or more powerful motor activation depending on the behavioral goals of the animal. While the advantage of greater inhibition of leech heart motoneurons through the HN inhibitory synapses is unclear currently, spike frequency in premotor neurons other motor CPGs has documented effects on the motor pattern (Katz and Frost, 1997; Brezina et al., 2003a,b). We can speculate that high spike frequency bursting will produce more prominent rebound and would be more effective in shaping the segmental motoneuron output pattern. In experiments where 1 μM myomodulin was applied to leech heart interneurons, there an increase in spike frequency ranging between 10 and 30% and reduction of the variability of the pattern (Tobin and Calabrese, 2005) was observed. While the difference in the co-existing regimes in the modulated HCO model is closer to 100%, the mere co-existence of these two particular regimes is novel. They burst on the same time scale, but spike very differently. To our knowledge, no multistability of this specific type has been identified in neural models. This unique form of multistabilty has interesting implications for the study of motor control by CPGs.

### 5.2 Downregulation of a current can result in larger average current

Another interesting result from this computational study is the observation that a cell model with a lower maximal pump current displayed a larger overall average pump current during the bursting cycle. This means that using an incomplete pharmacological inhibitor does not guarantee a reduction in the current, especially if steady state activity changes. It entirely depends on the dynamics of the other channels involved, which is often unknown in pharmacological experiments on neural circuits. This understanding should be applied to other systems in which the pump is modified. Downregulation or partial blocking of the pump does not guarantee that there will be less pump current. In fact, under certain conditions, it can increase the average pump current.

### 5.3 Neuroprotective effects of synaptic inhibition

While seizure activity in vertebrates cannot be assigned to a simple change in excitatory/inhibitory balance, general lack of network inhibition is often implicated (Tasker and Dudek, 1991; Calcagnotto et al., 2005; Cossart et al., 2005; Righes Marafiga et al., 2021). There can be no doubt, moreover, that bicuculline and other pharmacological agents that disrupt GABA-A transmission can cause seizures *in vivo* and *in vitro* (see for example Chitolina et al., 2023). In leech heart interneurons in intact ganglia, prolonged bicuculline exposure causes seizure like bursts (Cymbalyuk et al., 2002), and blocking of all chemical synaptic transmission (but not electrical) with Co^+2^ causes widespread synchronous seizure like bursts including heart interneurons (Angstadt and Friesen, 1991). Here we show that modulation involving the down-regulation of the Na^+^/K^+^ pump can lead to seizure-like activity in HN single cell and HCO models. This is not wholly surprising since the pump contributes outward current at all membrane potentials which is functionally equivalent to synaptic inhibition. Indeed Angstadt and Friesen (1991), provide evidence that the pump is critical to termination of seizure-like bursts induced by Co^2+^ and others have noted the importance of the pump during seizure activity (see for example Krishnan et al., 2015) Along the functional path of modulation of the HCO leading to smooth changes in rhythm period, modulation of the single component cells leads to seizure-like dysfunctional activity. Downmodulation of the pump current ultimately leads to a depolarization block in the single cell, but this is compensated through the synaptic inhibition inherent in the half-center circuit configuration. Cells which always receive some level of inhibition are tuned to function with it, and this dependence on inhibition for stability is one mechanism, among many, which can explain aberrant activity when inhibition is removed. Where one cell is incapable of bursting in a biological parameter range, a network of cells can robustly produce proper activity. A similar idea is discussed in prior work from our research group where the half-center oscillator structure can prevent the effects of an enhanced leak current from disrupting the pattern of activity in leech HN interneurons (Cymbalyuk et al., 2002). Thus, inhibitory circuits may be inherently more robust to modulation than their components neurons.

## Data availability statement

The raw data supporting the conclusions of this article will be made available by the authors, without undue reservation.

## Author contributions

PE: Conceptualization, Formal analysis, Investigation, Methodology, Visualization, Writing – original draft, Writing – review & editing, Software. YS: Writing – review & editing, Formal analysis, Investigation, Visualization. JP: Formal analysis, Investigation, Visualization, Writing – review & editing. RC: Funding acquisition, Methodology, Writing – original draft, Writing – review & editing. GC: Conceptualization, Data curation, Formal analysis, Funding acquisition, Investigation, Methodology, Project administration, Resources, Supervision, Validation, Visualization, Writing – original draft, Writing – review & editing.
