# Supplementary material for: Multistability of bursting rhythms in a half-center oscillator and the protective effects of synaptic inhibition
Source: Front Cell Neurosci. 2024 Sep 17;18:1395026. doi: 10.3389/fncel.2024.1395026 (PMC11442309; doi:10.3389/fncel.2024.1395026)
Supplement: Supplementary file 1 [file Table_1.docx]

Supplementary material 1 (S1)

S1: Table 1. Initial conditions for the 15-dimensional Single HN model.

|  | State variable | value |
| --- | --- | --- |
| HN | V | -0.011449235195089 V |
|  | m_CaF_ | 0.997488544633355 |
|  | h_CaF_ | 0.002707349837641 |
|  | m_CaS_ | 0.968605540692075 |
|  | h_CaS_ | 0.271653353056226 |
|  | m_K1_ | 0.873706094586109 |
|  | h_K1_ | 0.691193085259829 |
|  | m_K2_ | 0.319940796521384 |
|  | m_KA_ | 0.936059797824076 |
|  | h_KA_ | 0.014989472034014 |
|  | m_h_ | 0.150710992878140 |
|  | m_P_ | 0.809909131561657 |
|  | m_NaF_ | 0.936858375998633 |
|  | h_NaF_ | 0.140636395834389 |
|  | [Na]_i_ | 0.018191070092122 M |
