# Supplementary material for: Multistability of bursting rhythms in a half-center oscillator and the protective effects of synaptic inhibition
Source: Front Cell Neurosci. 2024 Sep 17;18:1395026. doi: 10.3389/fncel.2024.1395026 (PMC11442309; doi:10.3389/fncel.2024.1395026)
Supplement: Supplementary file 2 [file Table_2.docx]

S2: Table 2. Initial conditions for the 40-dimensional HCO model.

|  | State variable | LSFB | HSFB |
| --- | --- | --- | --- |
| HN(R) | V (R) | 0.002303511967636 V | 0.002937392198105 V |
|  | m_CaF_ (R) | 0.945097650744688 | 0.879356339228905 |
|  | h_CaF_ (R) | 0.043494617541268 | 0.213132016863981 |
|  | m_CaS_ (R) | 0.571062501235068 | 0.374306091669368 |
|  | h_CaS_ (R) | 0.387580790591235 | 0.614904285389434 |
|  | m_K1_ (R) | 0.649408345078278 | 0.677799248996130 |
|  | h_K1_ (R) | 0.911554562145083 | 0.941083132313783 |
|  | m_K2_ (R) | 0.174707109000414 | 0.150135359914835 |
|  | m_KA_ (R) | 0.829001606599812 | 0.814652820110204 |
|  | h_KA_ (R) | 0.036186984550938 | 0.054562210202260 |
|  | m_h_ (R) | 0.596490690510512 | 0.725710181587691 |
|  | m_P_ (R) | 0.686009887259014 | 0.669566696361869 |
|  | m_NaF_ (R) | 0.990804443112051 | 0.991716291189911 |
|  | h_NaF_ (R) | 0.425428570260358 | 0.421763292434286 |
|  | [Na]_i_ (R) | 0.017773651616569 M | 0.017715892129576 M |
|  | P (R) | 0.000000000004928 | 0.000000000003339 |
|  | A (R) | 0.000000000007782 | 0.000000000005886 |
|  | X (R) | 0.000000000000022 | 0 |
|  | Y (R) | 0.000138403796604 | 0.000000000000030 |
|  | M (R) | 0.524617233408450 | 0.302938462589456 |
| HN(L) | V (L) | -0.041507834761977 V | -0.076411916661275 V |
|  | m_CaF_ (L) | 0.962494342895857 | 0.000001495229571 |
|  | _hCaF_ (L) | 0.005411372523712 | 0.335330577260198 |
|  | m_CaS_ (L) | 0.945570786410148 | 0.000005527763042 |
|  | h_CaS_ (L) | 0.196214078072074 | 0.623185896476007 |
|  | m_K1_ (L) | 0.052005842709046 | 0.000419821402995 |
|  | h_K1_ (L) | 0.736702322682083 | 0.766520188761848 |
|  | m_K2_ (L) | 0.226662315560907 | 0.055289046898584 |
|  | m_KA_ (L) | 0.587020731591107 | 0.017703563897721 |
|  | h_KA_ (L) | 0.028060894155515 | 0.843691565503240 |
|  | m_h_ (L) | 0.199213039627756 | 0.286145228904327 |
|  | m_P_ (L) | 0.429053287495246 | 0.131050547053781 |
|  | m_NaF_ (L) | 0.132946379896650 | 0.000815155360709 |
|  | h_NaF_ (L) | 0.996361707534227 | 0.999999999794204 |
|  | [Na]_i_ (L) | 0.018433188573130 M | 0.019594879931725 M |
|  | P (L) | 0.000000000008767 | 0.000000000003706 |
|  | A (L) | 0.000000000016382 | 0.000000000007824 |
|  | X (L) | 0.619585875182626 | 0.642186768219535 |
|  | Y (L) | 0.059768404540586 | 0.066048673908143 |
|  | M (L) | 0.183921570228586 | 0.166422827570172 |
